# Feminizing hormone therapy using GnRH agonists as antiandrogens is not associated with adverse metabolic and bone effects in adult transgender women

**DOI:** 10.3389/fendo.2026.1725564

**Published:** 2026-06-12

**Authors:** Louise Brunet, Kanetee Busiah, Mireille Moser, Georgios E. Papadakis

**Affiliations:** 1 Faculty of Biology and Medicine, Lausanne University, Lausanne, Switzerland; 2 Pediatric Endocrinology Unit, Department of Pediatrics, Lausanne University Hospital, Lausanne, Switzerland; 3 Clinical Research Center of Lausanne University Hospital, Lausanne, Switzerland; 4 Department of Endocrinology, Diabetology and Metabolism of Lausanne University Hospital, Lausanne, Switzerland

**Keywords:** AMAB individuals, body composition, bone mineral density, estradiol, feminizing hormone therapy, GnRH analogs

## Abstract

**Introduction:**

Gender-affirming hormone therapy (GAHT) effectively alleviates gender dysphoria. However, its effect on metabolic and bone health is not well-established. The goal of this retrospective longitudinal study was to evaluate the effects of standardized feminizing GAHT using GnRH agonists and estrogens on metabolic and bone parameters in assigned male at birth (AMAB) individuals.

**Methods:**

Clinical and biochemical data of 40 AMAB individuals were collected at baseline, 6, 12, and 24 months of GAHT. A Dual X-ray absorptiometry to assess bone mineral density (BMD) and body composition was done at 12 months on GAHT. Primary outcomes included changes in BMI and body composition indexes. Potential predictors such as age and BMI at baseline, estradiol (E2) route, delta E2 rise, as well as the obtention of plasma E2 concentrations target (> 300 pmol/L), were explored.

**Results:**

Body mass index, fat mass index and visceral adipose tissue did not change (*p* = 0.14; *p* = 0.14; *p* = 0.73, respectively) under GAHT. However, GAHT resulted in a reduction in lean mass indices (-0.66 kg/m2, 95% CI [-0.96, -0.36], *p* < 0.001) at 12 months of treatment. Further, GAHT led to a consistent increase in plasma HDL-cholesterol (0.18 mmol/L, 95% CI [0.12, 0.24], *p* < 0.001). At baseline, 41% of participants had low BMD (Z-score < -2.0 SD); this declined to 27% by 12 months, accompanied by an increased BMD at lumbar spine [+0.03 g/cm^2^, 95%CI (0.02, 0.05), *p*=<0.001]. A greater E2 rise, as well as the attainment of the target of > 300 pmol/l for plasma E2 concentrations were not significantly associated with changes in metabolic outcomes at 12 months follow-up.

**Conclusions:**

Despite prior concerns, GAHT did not adversely affect surrogate markers of cardiovascular and bone health.

## Introduction

Transgender and gender-diverse (TGD) people are individuals who were assigned female at birth (AFAB) or male at birth (AMAB) but identify themselves to a different gender. They often experience gender dysphoria, which is defined by a distress and an uneasy feeling caused by the non-congruence between the designated gender and the gender identity (1). Gender-affirming hormone therapy (GAHT) is frequently prescribed to TGD individuals in order to induce secondary sex characteristics (e.g., body hair, breast or fat distribution) of the desired gender and thus to reduce gender dysphoria (2). Feminizing GAHT consists of increasing the estrogen blood concentration by taking exogenous estrogens, notably estradiol (E2) via oral or transdermal route (gel or patch). In addition, transgender women are offered antiandrogen therapy, which can be subcutaneous injections of GnRH agonists (GnRHa) or oral agents such as spironolactone or cyproterone acetate (CPA). To guide GAHT adjustments, Endocrine Society guidelines suggest targeting plasma E2 concentrations in the mid-follicular range for cis-women, a weak recommendation based on low evidence (1).

Besides physical appearance, sex steroid modulation via GAHT can also affect metabolism and bone. It is well established that both testosterone (T) and E2 are crucial regulators of metabolic homeostasis (3, 4), as well as powerful stimulators for bone mineral density (BMD) accrual in both sexes (5). Available studies to date have shown conflicting findings regarding the impact of GAHT on metabolic and bone health. For example regarding lipid profile, a longitudinal study in the United States detected a small but significant improvement in HDL-cholesterol levels in of 170 transgender women (6), but this finding was not reproduced in a subsequent metanalysis (7). Similarly, an increase to bone mineral density (BMD) was reported by a metanalysis of nineteen available studies (8), in contrast to a more recent review which did not objectify any consistent change in bone parameters (9). Significant heterogeneity of GAHT protocols across different studies, as well as possible lack of compliance with therapeutic targets may have rendered definitive conclusions more difficult.

We, thus, aimed herein to evaluate the change in clinical and biochemical markers of metabolic and bone health in AMAB individuals over specific timepoints following the initiation of a standardized GAHT regimen implementing GnRHa and for two years thereafter. Factors such as age at GAHT initiation, baseline body mass index (BMI), E2 administration route, and achieved concentrations of reproductive hormones during GAHT were analyzed as potential predictors of metabolic and bone outcomes.

## Methods

### Study design and population

This was a retrospective longitudinal study conducted at the EDM service of CHUV. The period of data collection was between 01.01.2018 and 31.10.2024. Potentially eligible patients were identified through an anonymized extraction process, which revealed 109 eligible patients. Subsequently, three additional patients were identified, bringing the total number of eligible patients to 112. Following approval of the study’s protocol by the Ethics Commission of the Canton of Vaud (CER-VD) in April 2023, eligibility among the 112 patients was determined.

Patients were eligible if they met all the following inclusion criteria: 1. Diagnostic of Gender Dysphoria in an AMAB individual between January 1^st^ 2018 and September 30^th^ 2022, followed at EDM service of CHUV; 2. Age ≥ 18 years at the beginning of gender affirming hormone therapy; 3. Use of GnRHa as anti-androgens within GAHT; 4. Once initiated, duration of Gender Affirming Hormone therapy (estrogen-based) for at least 6 months; 5. Signature of a specific consent for this study or having agreed on reuse of clinical data for research purpose by signing the general consent of the CHUV. Exclusion criteria included: 1. Discontinuation of Gender Affirming Hormone therapy for > 3 months during follow-up; 2. Uncontrolled eating disorders; 3. New onset of medications (e.g., exogenous glucocorticoids, neuroleptics) or medical conditions known to promote weight gain, if they were not present at baseline.

**Figure 1:**
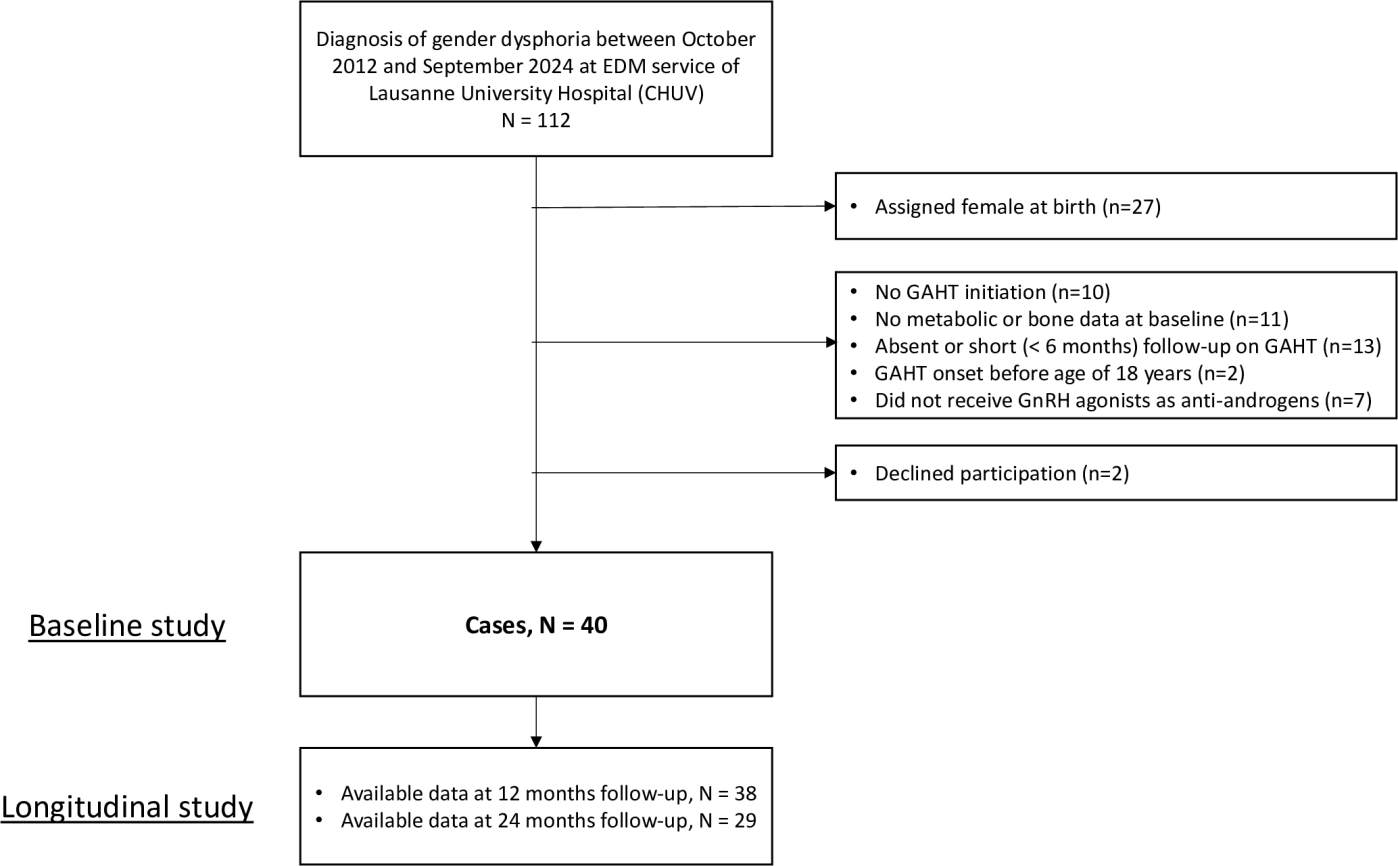
Flow chart of the study. The selection process of participants is shown. GAHT, Gender-affirming hormone therapy.

Out of the 112 potentially eligible patients, 42 met the inclusion criteria. Specific consent was obtained from those who had not signed the CHUV’s general consent, resulting in 40 patients who were both eligible and provided consent. A flow chart illustrating the selection process is shown in **Figure 1**.

### GAHT protocol

GAHT consisted of the administration of GnRH agonists in combination with estrogens according to a standardized protocol. The GnRH agonists used was Leuprolide acetate (Lucrin Depot^®^, AbbVie AG), administered as a 3.75 mg subcutaneous injection once per month for the first three months, followed by shift to the 11.25 mg form every three months. If target testosterone levels (<1.4 nmol/L) were not achieved, the injection interval was shortened to every 10 weeks and subsequently to every 8 weeks if needed. Estrogens could be administered via oral, transdermal gel or transdermal patch. Oral E2 (Estrofem^®^, Novo Nordisk Pharma AG) was initiated at 2 mg daily and subsequently adjusted every three months to achieve target plasma levels (> 300 pmol/L). Transdermal estradiol gel (Oestrogel^®^, Vifor (International) Inc.) was initiated at two pushes daily (1 push = 0.75 mg), with dose adjustments performed similarly. Alternatively, estradiol patches (Estradot^®^, Sandoz Pharmaceuticals AG) were prescribed at 75 µg (50 µg in individuals aged >40 years), to be changed every three days, with dose adjustments as needed to achieve the same plasma estradiol levels targets. Patients were followed every three months during the first year and every six months during the second year of the follow-up.

### Data collection

At the EDM service, a standardized management protocol was established following publication of 2017 Endocrine Society guidelines. Subsequently, all patients undergo a standard baseline assessment before the beginning of GAHT, which includes a clinical examination, a fasting blood test and a whole-body Dual X-Ray absorptiometry (DXA scan) for body composition analysis and bone densitometry. The follow-up consists of a clinical consultation and a blood test every 3 months during the first year, then every 6 months during the second year of GAHT. The dose of estrogen and anti-androgen therapy was adjusted to target plasma T and E2 concentrations in the AFAB range in line with the Endocrine Society guidelines (1) and more recently with the World Professional Association for Transgender Health guidelines (10). In addition, patients undergo a DXA scan for bone densitometry and body composition 12 months after GAHT. Relevant clinical and biochemical data were extracted from medical files of participants in a secured coded manner. More specifically, we retrieved data at baseline, 6 (M6), 12 (M12) and 24 (M24) months of follow-up, along with relevant medical history.

Estrogen route administration was selected based on the patient’s preference. For patients aged > 45 years or with risk factors for thromboembolic disease (i.e., positive family history, smoking, obesity), a transdermal estrogen route was recommended but not imposed. Regarding anti-androgen therapy, all patients received GnRHa, which is the first-line choice in our department since 2018, because of easy application in our outpatient clinic and eviction of compliance issues. Importantly, none of the patients had received prior GnRHa therapy for pubertal suppression.

### DXA scan

DXA scans (Lunar iDXA, GE Healthcare) for BMD measurement and body composition assessment was performed in our department as previously described (11, 12). Results were interpreted in accordance with the recommendations of the International Society for Clinical Densitometry (https://iscd.org/learn/official-positions/). Given that the majority of participants were aged < 40 years, we used Z-scores to define low BMD as Z-score < -2.0 DS at least in one of the following sites: Lumbar spine (LS), Femoral Neck (FN) and/or Total Hip (TH). As the baseline DXA scan was performed before GAHT initiation, we calculated Z-scores based on age, ethnicity and sex assigned at birth. At the follow-up DXA assessment (1 year after GAHT initiation), the administrative sex of the patient was used for the reference population which was female for the large majority of cases. This choice remained in accordance with the ISCD position for DXA measurements in transgender populations (13). To avoid the bias due to the overestimation of Z-scores in the follow-up DXA scan, we focused mainly on the longitudinal changes of absolute BMD shifts (in g/cm^2^). Regarding body composition, the main derived outcomes were fat mass index (FMI), lean mass index (LMI) and appendicular lean mass index (ALMI) (14). Visceral adipose tissue (VAT) was measured as the fat tissue located deep in the abdomen around the internal organs, as opposed to subcutaneous adipose tissue.

### Biochemical assessment

Blood tests at baseline and follow-ups were performed in the morning after overnight fasting. The following data were collected: (i) Hormones: E2, T, Sex-Hormone binding globulin (SHBG), Luteinizing hormone (LH), Follicle stimulating hormone (FSH) and prolactin; (ii) Metabolic parameters: Fasting blood glucose (FBG), Glycated hemoglobin (HbA1c), Triglycerides (TG), High density lipoprotein cholesterol (HDL-Cholesterol), Low density lipoprotein cholesterol (LDL-Cholesterol), Alanine transaminase (ALAT), High sensitive C-reactive protein (HsCRP); (iii) Bone parameters: P1NP and β-Crosslaps.

All biological measurements were performed by the Lausanne University Hospital Clinical Laboratory using commercially available immunoassays (mainly Cobas, Roche Diagnostics) on fresh blood samples within 2 hours of blood collection. Extreme values of E2 were removed in three visits due to plausible contamination during sample collection, attributable to transdermal mode of E2 administration.

### Outcomes and potential predictors

The principal outcome was changes in BMI as well as indexes of body composition with link to metabolic health (FMI, ALMI, VAT). Shifts in bone parameters such BMD_LS, BMD_FN and BMD_TH, as well as in trabecular bone score (TBS) served as secondary outcomes. Additional outcomes included changes in glucose metabolism, lipid profile, liver enzymes and low-grade inflammation.

We preselected as potential predictors (co-variables) to explore the following parameters: age at GAHT initiation, pre-GAHT BMI, route of E2 administration in the GAHT regimen, as well as the induced rise (delta) in E2 concentrations from baseline to 12 months (M12) of GAHT. We also investigated an additional binary modifier: whether the lower target concentration of E2 plasma concentrations (≥300 pmol/L) was reached by M12 or not. This target has been proposed as it corresponds to the physiological mid-follicular peak of E2 in cis-gender women (1).

### Statistical analysis

Differences between time points were estimated by a mixed-effects regression with time (factor) as a fixed effect, and a patient-specific random effect. An unstructured within group correlation (time) was considered. Due to convergence issue, the mixed-effects model for BMI could not be reliably estimated. We therefore used a paired t-test to compare BMI between the timepoints. The effect of delta in E2 and rise of the threshold was estimated by linear regression adjusted for baseline value, root of administration of GAHT_E2, BMI and age at GAHT initiation. Missing data were considered missing at random. Multiple imputations were performed for both mixed-effects and linear regressions. Missing data were imputed using available information of the same measurement at other time points. For all DXA variables (FMI, LMI, VAT and ALMI), BMI was also accounted for. Multiple imputations were performed using the mice package (version 3.16.0) (15).

All statistical analysis were performed with the R software (version 4.3.0). The following R version was used: R version 4.3.0 (2023-04–21 ucrt) (16).

## Results

Our cohort of 40 AMAB patients had a mean age of 29 years old. Details on E2 route of administration are displayed in **Table 1**. Roughly 60% of the patients opted for transdermal estrogens.

**Table 1:** Age, type of GAHT and Z-scores of bone mineral density at baseline.

Parameters of interest	Results
Age (years)	29.2 ± 12.2
E2 route of administration	
Oral	18/40 (45)
Transdermal (gel or patch)	22/40 (55)
Bone mineral densities Z-scores	
BMD_LS	-1.6 ± 1.44
BMD_TH	-0.87 ± 0.86
BMD_FN	-0.84 ± 0.86

The data are shown as the mean ± standard deviation. Qualitative parameters are shown as N/total (percentage). E2, estradiol. BMD, Bone mineral density. LS, Lumbar spine. TH, Total hip. FN, Femoral neck.

The evolution of hormone concentrations during the study period is provided in **Table 2**. Detailed available sample size for each parameter over different timepoints is displayed in **Supplementary Table 1** of Appendix. As intended, estradiol increased from a mean of 110 ± 50 pmol/L at baseline to 310 ± 230 pmol/L at the 24 months follow-up. Nevertheless, the percentage of participants who achieved the plasma E2 goal varied between 34% and 47% in different timepoints (**Supplementary Table 1**). The estrogen rise was accompanied by an increase in plasma SHBG concentrations already at M6 [23 nmol/L, 95% CI (16.03, 29.96), *p* < 0.001] and stable thereafter. Testosterone concentrations dropped sharply to less than 2 nmol/L at both M12 and M24 in most individuals. Given that all patients received GnRHa, LH and FSH decreased significantly at M6 [LH: -5.11 IU/L, 95% CI (-6.83, -3.39), *p* < 0.001; FSH: -5.03 IU/L, 95%CI (-7.61, -2.44), *p* < 0.001] and M12 [LH: -5.88 IU/L, 95% CI (-7.29, -4.46), *p* < 0.001; FSH: - 5.76 IU/L, 95%CI (-8.27, -3.25), *p* < 0.001]. At M24, mean concentration of both LH and FSH were higher, because of the fact that three patients underwent gonadectomy in the meantime and therefore discontinued GnRHa. GAHT had no significant effect on plasma prolactin concentrations.

**Table 2:** Evolution and comparison between timepoints of reproductive hormones.

Hormones	Baseline	M6	M12	M24	P value M6 vs BL	P value M12 vs BL	P value M24 vs BL
Estradiol (pmol/L)	100 (80, 130)	260 (160, 320)	290 (170, 450)	260 (170, 420)	**<0.001**	**<0.001**	**<0.001**
Testosterone (nmol/L)	17.2 (14.1, 22.8)	1.0 (0.8, 1.5)	1.0 (0.8, 1.2)	1.1 (0.8, 1.2)	**<0.001**	**<0.001**	**<0.001**
SHBG (nmol/L)	27 (21.5, 39)	44 (34, 71)	53.5 (38, 74)	50 (36, 64.5)	**<0.001**	**<0.001**	**<0.001**
LH (IU/L)	6.1 (4.1, 7.1)	1 (0.5, 1.5)	0.6 (0.5, 1.2)	0.6 (0.4, 1.7)	**<0.001**	**<0.001**	0.76
FSH (IU/L)	4.0 (2.9, 6.2)	0.5 (0.2;1.1)	0.4 (0.2, 0.8)	0.5 (0.2, 1.6)	**<0.001**	**<0.001**	0.76
Prolactin (µg/L)	12.8 (7.5, 17.3)	15 (9.2, 19.0)	12.4 (10.3, 16.7)	12.7 (9.6, 20)	0,729	0,966	0.28

The data are shown as median (Q25, Q75). SHBG, Sex hormone-binding globulin. LH, Luteinizing hormone. FSH, Follicle stimulating hormone. Significant differences (P value < 0.05) are highlighted in bold.

**Figure 2:**
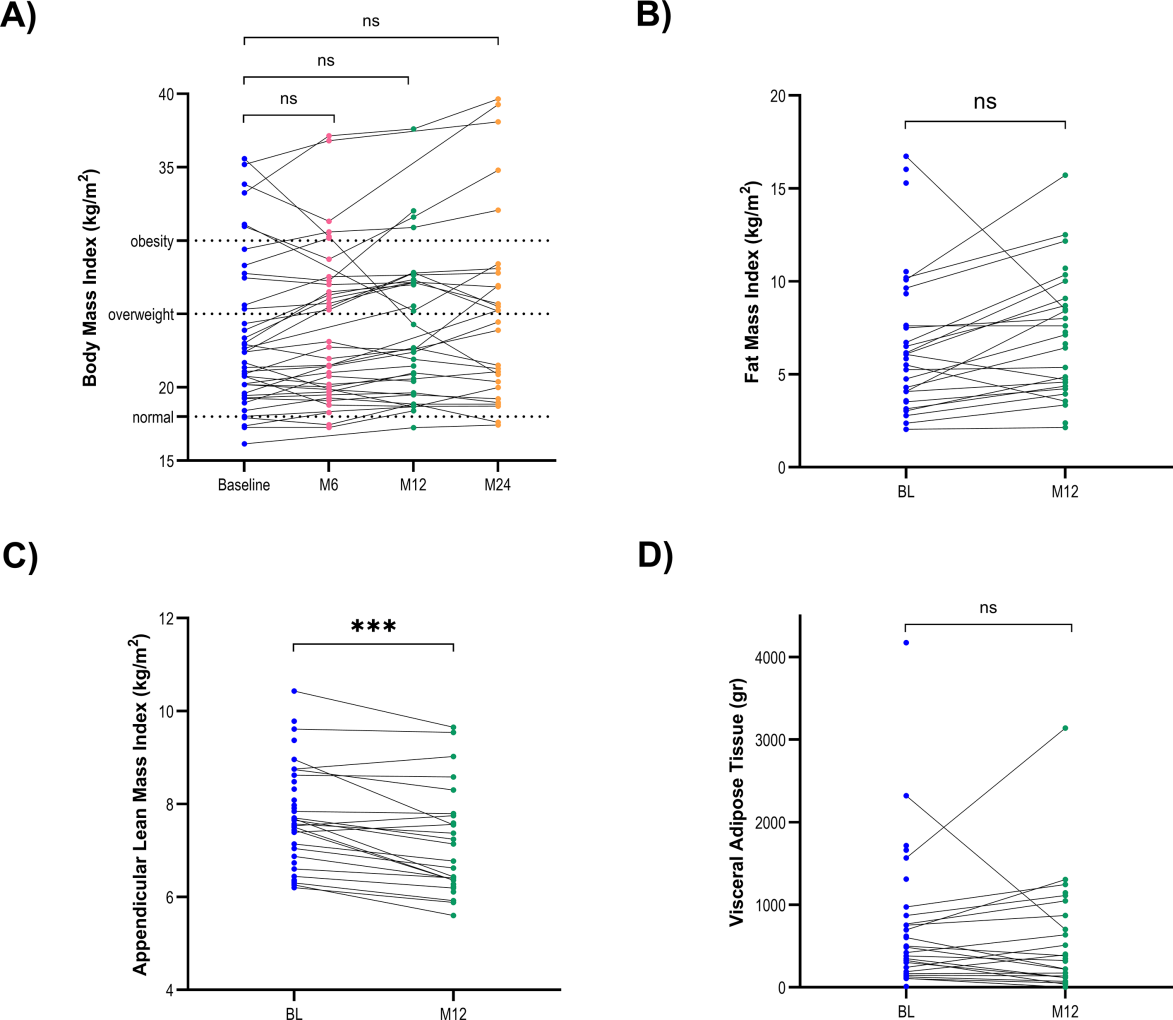
Change in anthropometrics and body composition under gender-affirming hormone therapy. **(A)** Changes in Body Mass Index. **(B)** Changes in Fat Mass Index under GAHT. **(C)** Changes un Appendicular Lean Mass Index. **(D)** Changes in Visceral Adipose Tissue. Each line corresponds to an individual patient. Data at baseline, Month 6, Month 12, Month 24 are shown in blue, pink, green and orange circles, respectively. In panel **(A)** dotted lines correspond to the lower limits for normal BMI, overweight and obesity. ***p<0.001; ns, not significant.

An overview of the evolution of metabolic markers and body composition parameters is shown in **Table 3**. BMI did not change significantly over time (*p* = 0.142), with the mean going from 23.4 at baseline to 25.3 kg/m^2^ at M24 (**Figure 2A**). Similarly, total fat mass, as expressed by FMI, showed no change across timepoints (from 6.84 kg/m^2^ at baseline to 7.04 kg/m^2^ at M12, *p* = 0.136) (**Figure 2B**). Importantly, no evidence of visceral fat accumulation was observed (*p* = 0.729, **Figure 2D**). Meanwhile, lean mass indexes (LMI and ALMI) decreased at 12 months (LMI: -0.66 kg/m2, 95% CI [-0.96, -0.36], *p* < 0.001, ALMI: -0.44 kg/m2, 95% CI [-0.63, -0.26], *p* < 0.001, **Figure 2C**). Regarding the biochemical metabolic parameters, HDL-cholesterol concentrations rose consistently over time (M6: +0.19 mmol/L, 95% CI [0.13, 0.26], *p* < 0.001; M12: +0.18 mmol/L, 95% CI [0.12, 0.24], *p* < 0.001; M24: +0.11 mmol/L, 95% CI [0.01, 0.20], *p* = 0.029) (**Table 3**). Other lipid concentrations as well as liver tests and serum high-sensitivity CRP concentrations remained stable.

**Table 3:** Evolution and comparison between timepoints of metabolic parameters and body composition.

Parameters of interest	Baseline	M6	M12	M24	P value M6 vs BL	P value M12 vs BL	P value M24 vs BL	
Metabolic parameters								
BMI (kg/m^2^)	23.4 ± 5.3	24.1 ± 5.2	23.7 ± 4.8	25.3 ± 6.5	0,209	0,135		0,142
FBG (mmol/L)	4.9 ± 0.5	4.8 ± 0.4	5.1 ± 0.5	5.0 ± 0.5	0,106	0,342		0,278
HbA1c (%)	5.2 ± 0.4	5.1 ± 0.4	5.1 ± 0.3	5.1 ± 0.4	0,124	0,199		0,235
HOMA-IR	2.72 ± 1.7	2.54 ± 1.38	2.63 ± 1.44	2.92 ± 1.68	0,521	0,842		0,588
Triglycerides	1.18 ± 0.72	1.2 ± 0.63	1.32 ± 0.72	1.17 ± 0.45	0,884	0,207		0,897
HDL-Cholesterol (mmol/L)	1.39 ± 0.35	1.57 ± 0.38	1.58 ± 0.39	1.51 ± 0.46	**<0.001**	**<0.001**		**0,029**
LDL-Cholesterol (mmol/L)	2.48 ± 0.91	2.45 ± 0.77	2.43 ± 0.89	2.62 ± 0.95	0,591	0,391		0,762
ALAT (UI/L)	26 ± 13	32 ± 25	29 ± 20	26 ± 10	0,061	0,333		0,8
HsCRP (mg/L)	1.7 ± 2.2	2 ± 3.6	1.7 ± 2.1	2.4 ± 4.1	0,312	0,633		0,158
Body Composition								
FMI (kg/m^2^)	6.84 ± 3.84	–	7.04 ± 3.33	–		0,136		–
VAT (gr)	700.56 ± 851.02	–	543 ± 649	–		0,729		–
LMI (kg/m^2^)	16.34 ± 2.13	–	15.41 ± 2.08	–		**<0.001**		–
ALMI (kg/m^2^)	7.75 ± 1.11	–	7.16 ± 1.11	–		**<0.001**		–

The data are shown as the mean ± standard deviation. BMI, Body mass index. FBG, Fasting blood glucose. HbA1c, Glycated hemoglobin. HOMA-IR, Insulin resistance score. ALAT, Alanine Aminotransferase. HsCRP, High sensitive C-Reactive Protein. FMI, Fat mass index. VAT, Visceral adipose tissue. LMI, Lean mass index. ALMI, Appendicular lean mass index. Significant differences (P value < 0.05) are highlighted in bold.

**Figure 3:**
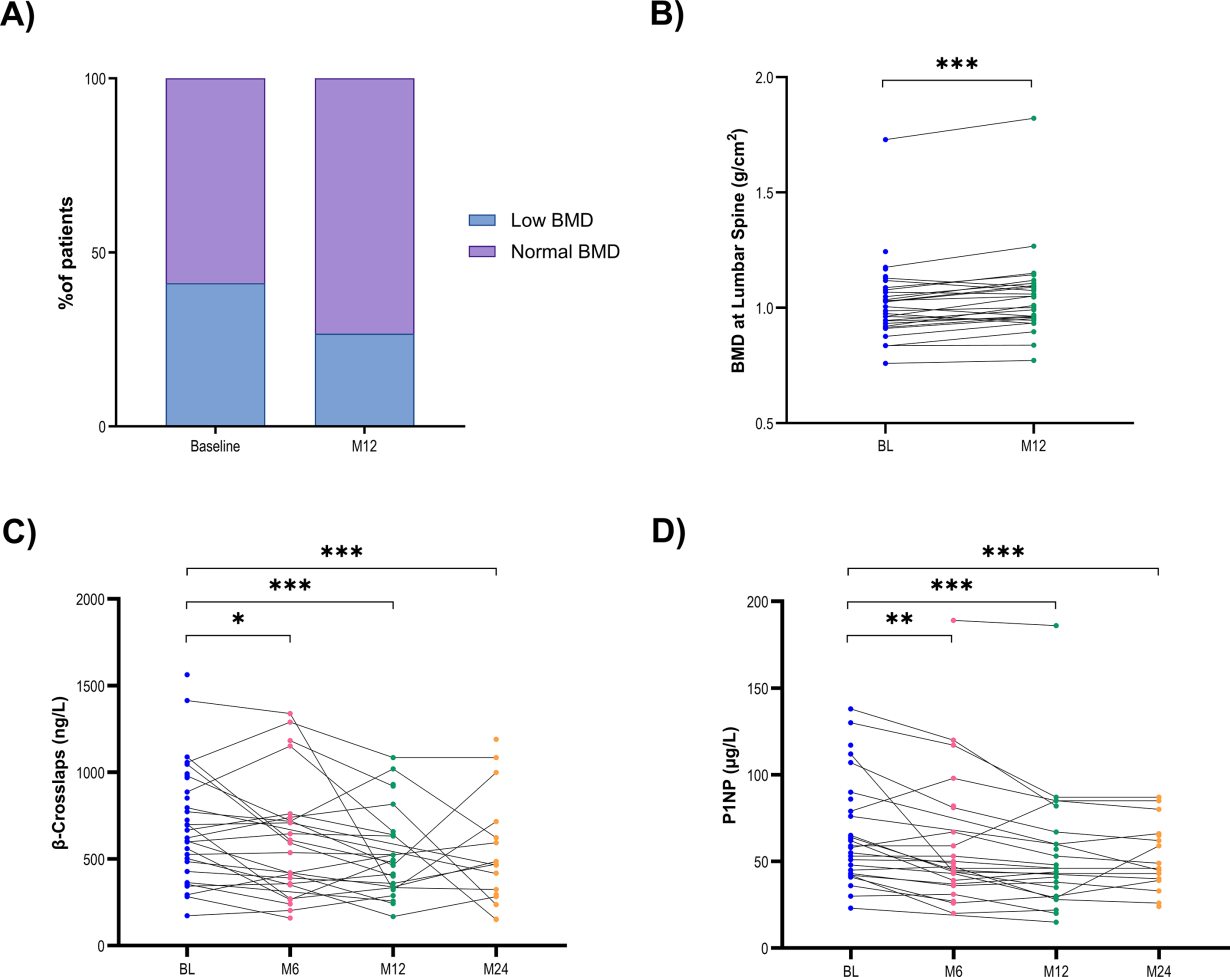
Effect of gender-affirming hormone therapy on bone density and serum remodeling markers. **(A)** Proportion of patients with low bone mineral density (BMD). **(B)** Changes of BMD at Lumbar Spine under GAHT. **(C)** Changes of β-Crosslaps under GAHT. **(D)** Changes of P1NP under GAHT. In panel **(A)** patients with low BMD are illustrated in light blue, as compared to those with normal BMD in purple. Each line corresponds to an individual patient. Data at baseline, Month 6, Month 12, Month 24 are shown in blue, pink, green and orange circles, respectively. ***p<0.001; **p<0.01; *p<0.05.

Bone health was studied through DXA-derived measurements of bone mass (BMD) and bone structure (trabecular bone score, TBS), as well as bone remodeling serum markers, β-crosslaps and P1NP, which estimate bone resorption and bone formation, respectively (17). Results are shown in **Table 4** and **Figure 3**. Low BMD (Z-score < -2.0 DS at one site at least) was prevalent at baseline (before GAHT initiation) occurring in 41% of participants. An improvement was noted at M12 (low BMD 27% of participants, **Figure 3A**), accompanied by a small increase of BMD at LS [paired difference: 0.03 g/cm^2^, 95% CI (0.02, 0.05), *p*=<0.001, **Figure 3B**], while BMD at femoral site and TBS did not show any considerable variation. The benefits in BMD were also reflected in a reduction of both β-crosslaps and P1NP (**Figures 3C, D**), which started at M6 [β-crosslaps: -84 ng/L, 95%CI (-165, -3), *p* = 0.042, P1NP: -11.04 µg/L, 95% CI (-17.74, - 4.34), *p* = 0.001] and progressively intensified by M24 [β-crosslaps: -239 ng/L, 95%CI (-361, -117), *p*=<0.001, P1NP: -17.13 µg/L, 95% CI (-26.34, -7.93), *p*=<0.001].

**Table 4:** Evolution and comparison between timepoints of bone parameters and bone remodeling.

Bone parameters	Baseline	M6	M12	M24	P value M6 vs BL	P value M12 vs BL	P value M24 vs BL
BMD_LS (g/cm^2^)	1.028 ± 0.166		1.045 ± 0.178	–	–	**<0.001**	–
BMD_TH (g/cm^2^)	0.959 ± 0.104		0.948 ± 0.105	–	–	0,823	–
BMD_FN (g/cm^2^)	0.955 ± 0.100		0.944 ± 0.108	–	–	0,862	–
Low BMD	14/34 (41)	–	8/30 (27)	–	–	0.29	–
TBS	1.45 ± 0.07		1.44 ± 0.07			0,169	
P1NP (µg/L)	66.23 ± 29.19	60.42 ± 38.33	54.59 ± 36.59	52.47 ± 19.36	**0,001**	**<0.001**	**<0.001**
β-Crosslaps (ng/L)	710.45 ± 325.15	598.17 ± 350.91	535.04 ± 262.17	523.71 ± 313.89	**0,042**	**<0.001**	**<0.001**

Data are shown as the mean ± standard deviation. BMD_LS, Bone mineral density of lumbar spine. BMD_TH, Bone mineral density of total hip. TBS, Trabecular bone score. Low BMD was defined as Z-score < -2.0 SD at one or more skeletal sites. For low BMD, data are shown as N/total (percentage). Significant differences (P value < 0.05) are highlighted in bold.

**Figure 4:**
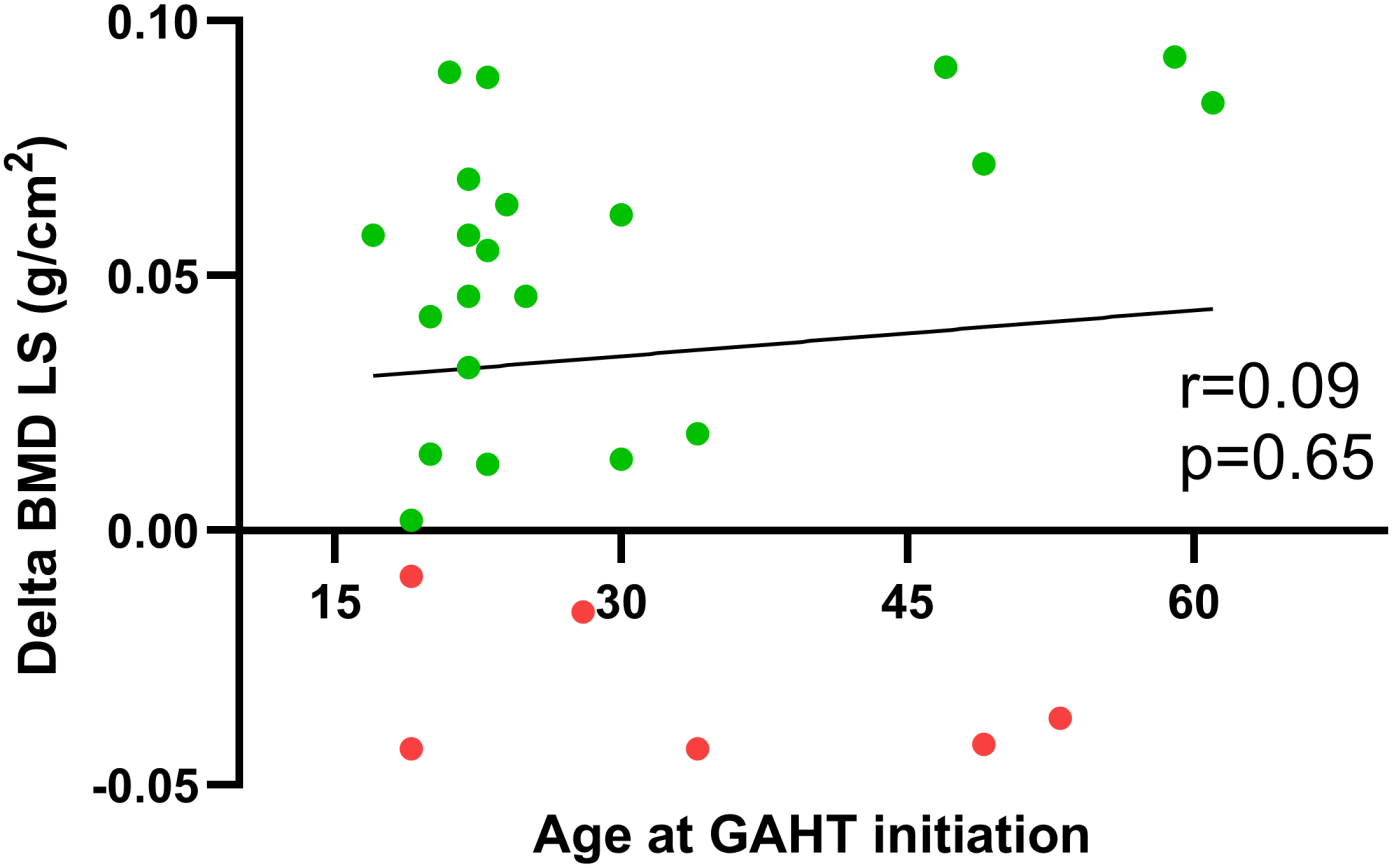
Univariate linear regression between age at GAHT initiation and delta BMD at lumbar spine. Each spots corresponds to an individual participant, green spots account for increases in BMD, red spots account for decreases in BMD.

Since a considerable portion of participants (23/40) initiated GAHT before the age of 25 years, which is traditionally considered as the age of acquisition of peak bone mass (18), we explored whether the magnitude of the effect of E2 on BMD could varied depending on age at GAHT initiation. A simple linear regression between age at GAHT initiation and the delta BMD change at lumbar spine did not show any significant association. (**Figure 4**). Similar results were found for the other bone outcomes (data not shown).

We then assessed the effect of predefined co-variables on the variation of metabolic and bone outcomes. After multivariate adjustment for age at GAHT initiation, BMI and TG at baseline, as well as for GAHT route administration, the degree of E2 rise by M12 was not associated with plasma TG concentrations at M12 (-0.09 mmol/l for each additional 100 pmol/l of delta E2 rise, 95% CI [-0.19, 0.01], *p* = 0.073, **Supplementary Figure 1**). Moreover, no significant associations were observed with BMI, FMI and VAT at M12 (**Supplementary Table 2**). The attainment of E2 plasma target of 300 pmol/l by M12 showed no significant associations with metabolic outcomes (**Supplementary Figure 1**; **Supplementary Table 3**).

## Discussion

Our longitudinal study allows to characterize the metabolic and bone imprint of feminizing GAHT in AMAB individuals using a standardized protocol with GnRH agonists as the sole anti-androgen therapy. Any eventual adverse metabolic effect of GAHT can translate into increased risk for cardiovascular morbidity and mortality. Notably, a recent systematic review and meta-analysis by Van Zijverden et al. (19) showed an increase in cardiovascular risk in AMAB individuals undergoing GAHT. However, most available data is based on cross-sectional studies, and it is thus unclear whether raised cardiovascular risk is due to preexisting health factors and a potential minority gap or to the metabolic side effects of GAHT itself.

Our data indicate that AMAB individuals treated with GnRHa-based GAHT do not experience any significant increase in BMI and total fat mass. More importantly, we did not detect any unfavorable change in VAT levels. In contrast, a significant decrease in lean mass was observed, presumably due to the underlying drastic reduction in T concentrations. This dual impact of GAHT on body composition has been previously reported (5). Nevertheless, only a couple of longitudinal studies have specifically looked at visceral fat and both of them used MRI and not the readily-available DXA as the imaging tool. Klaver et al. (20) studied 179 transgender women treated with CPA and found stable VAT despite an increase in total fat. A smaller cohort of 16 transgender women also exhibited stable VAT, as assessed by MRI, before and after a GnRHa-based GAHT regimen (21). Although important inter-individual variability was observed in VAT range, our finding of a neutral effect in a larger GnRHa-treated cohort despite the strong inhibition of the endogenous hypothalamus-pituitary-testicular axis is reassuring.

The changes in metabolic blood markers were also reassuring from a cardiovascular perspective. HDL-cholesterol concentrations consistently increased with GAHT. This favorable shift was likely due to a direct effect of estrogens on lipoprotein metabolism, as suggested by Haddock et al. (22) who showed a similar increase in HDL-cholesterol with post-menopausal hormone replacement therapy. Other lipid markers, as well as blood sugar, liver function and low-grade inflammation remained stable in our cohort. Prior literature is marked by striking between-studies differences regarding the effect of GAHT on lipid profile in transgender women, possibly due to several confounding factors, such as the type of antiandrogen therapy, the degree of E2 rise and the duration of follow-up. A recent large cohort reported a decrease in all lipid profile parameters (including HDL-cholesterol) after 1 year of GAHT (20). However, patients in that study were treated with CPA. Further, E2 concentrations only mildly rose to a mean of 200 pmol/L during the follow-up period. In contrast, Leemaqz et al. (6) using mostly spironolactone as antiandrogen therapy observed a decrease in LDL-Cholesterol, an increase in HDL-Cholesterol with TG and TC remaining stable after more than 24 months of GAHT. Our study highlights the lipid signature of a GnRHa GAHT regimen, which is lacking in the current literature. This is also evident when comparing our results to two recent meta-analysis (7, 23), which mostly incorporated studies using CPA or spironolactone.

In search of the optimal GAHT regimen to favor metabolic health, we investigated several potential modifiers, focusing on their effect on metabolic outcomes at 12 months of follow-up. Our hypothesis that transdermal estrogen route administration would be associated with a better metabolic response was not confirmed. This finding is in line with a systematic review and meta-analysis by Miranda et al. (24), although some bias (older and more obese transgender women are offered preferentially transdermal E2) cannot be excluded. Moreover, our study did not provide convincing proof that a more efficient rise in estrogen concentration (delta-E2 from baseline to M12) or the attainment of target plasma E2 concentration are linked with a more favorable metabolic profile. While no significant association was found, further investigation within larger cohorts with higher degree of successfully reached E2 plasmatic targets is needed. In the setting of a drastic reduction of T concentrations to the cisgender female range (induced by GnRHa), it is plausible to hypothesize that effective sex steroid replacement with E2 may still contribute to avoid adverse metabolic effects. Indeed, uncompensated testosterone deficiency is an established risk factor for weight gain, visceral fat accrual and metabolic dysfunction in cisgender men (25).

Transgender women in our study exhibited high incidence of low BMD despite relatively young age and mean BMI in the higher normal range. Previous observational studies also reported low BMD in roughly 30% of transfeminine individuals (26, 27). Despite this alarming baseline data, our results delineate an absence of negative impact on bone homeostasis, as evidenced by a minor but significant increase in the BMD at lumbar spine at 12 months, as well as a deceleration of bone turnover as the presumed underlying mechanism. A recent observational study also reported improvements in BMD at lumbar spine after 12 months (28). The preferential BMD gain at the lumbar spine as opposed to the hip is likely due to the trabecular-rich content of the former, rendering it more prone to sex steroid-mediated effects (29). E2 is moreover recognized as a major sex hormone that prevents bone loss in both men and women (8), suggesting that feminizing GAHT is likely to not produce any negative effects provided that adequate E2 levels are achieved. These findings align with results of several studies (8, 28, 30). However, in Ceolin et al. (28), the type of GAHT was not specified, while in Fighera et al. (8) GAHT regimens predominantly used cyproterone acetate. Similarly, in the only available meta-analysis on bone effects of GAHT by Singh-Ospina et al. (30), the included studies involved different types of GAHT, the majority of whom implemented CPA as antiandrogen. Adding to current state of knowledge, our data indicate that GAHT regimens using mostly GnRHa as antiandrogens are not associated with adverse bone effects despite more drastic reduction in T concentrations.

Our work has several limitations. Firstly, our sample size was relatively small. Our results regarding the role of E2 change on metabolic outcomes should thus be considered exploratory and require further exploration in larger studies with higher attainment of the targeted plasma levels. In this one-arm observational study we were not able to perform a head-to-head comparison of different estrogen dose and antiandrogen classes. Due to the retrospective design, we encountered missing data in some outcomes/co-variables. However, statistical treatment was applied under the clinically relevant hypothesis of missing at random. Missing values increase the uncertainty on final estimates, resulting in sometimes large confidence intervals. This was a single-center study in mostly Caucasian individuals, limiting in part the generalizability of our findings. Lastly, we did not evaluate some potential confounding factors that could have indirectly affected metabolic and bone health such as physical activity status, mental health status and vitamin D concentrations.

On the contrary, our study displays significant strengths. Participants were followed based on a standardized protocol in accordance with Endocrine Society Guidelines. Consequently, our cohort exhibited good homogeneity in terms of therapeutic management and the use of GnRHa as the antiandrogen of choice. The latter was important due to the relative dearth of data with this antiandrogen class as compared to CPA and spironolactone. Bone mineral density was measured with DXA, a gold-standard tool. The latter is also a validated reference method to assess body composition. Lastly, in a field with low-evidence recommendations, we proposed a novel approach by examining GAHT-induced hormonal changes as potential predictors of subsequent metabolic response, a first step toward a more personalized approach.

In conclusion, our longitudinal study provides several reassuring lines of evidence in favor of the metabolic and bone security of feminizing GAHT in AMAB individuals. These results are specific to a GAHT regimen with GnRH agonists as anti-androgen. Taken together, these data suggest that the elevated cardiovascular and fracture risk in transfeminine individuals, which is found in epidemiological studies, seems to be preexistent to GAHT and not caused by it. Given that both the current and previous studies have mostly assessed surrogate markers, large multicentric prospective studies focusing on hard outcomes (cardiovascular events, fractures) in well-defined and standardly-treated TGD cohorts are required.

## Data Availability

The raw data supporting the conclusions of this article will be made available by the authors, without undue reservation.
