# Supplementary material for: Feminizing hormone therapy using GnRH agonists as antiandrogens is not associated with adverse metabolic and bone effects in adult transgender women
Source: Front Endocrinol (Lausanne). 2026 Jun 12;17:1725564. doi: 10.3389/fendo.2026.1725564 (PMC13303374; doi:10.3389/fendo.2026.1725564)
Supplement: Supplementary file 1 [file DataSheet1.docx]

**Supplementary Table S1**. Sample size for each parameter of the longitudinal analysis

|  | **Baseline** | **M6** | **M12** | **M24** |  |
| --- | --- | --- | --- | --- | --- |
| Reproductive hormones |  |  |  |  |  |
| Estradiol | 39 | 38 | 35 | 28 |  |
| Estradiol goal reached (>300pmol/L) | - | 13/38 (34) | 17/36 (47) | 9/28 (32) |  |
| Testosterone | 36 | 38 | 31 | 25 |  |
| SHBG | 39 | 28 | 32 | 25 |  |
| LH | 39 | 38 | 34 | 28 |  |
| FSH | 39 | 38 | 34 | 28 |  |
| Prolactin | 38 | 17 | 26 | 24 |  |
| Metabolic parameters |  |  |  |  |  |
| BMI | 40 | 35 | 35 | 28 |  |
| Fasting blood glucose | 40 | 38 | 33 | 27 |  |
| HbA1c | 32 | 28 | 31 | 25 |  |
| HOMA-IR | 32 | 30 | 28 | 26 |  |
| Triglycerides | 40 | 38 | 37 | 27 |  |
| HDL-Cholesterol | 40 | 38 | 37 | 27 |  |
| LDL-Cholesterol | 40 | 38 | 37 | 26 |  |
| ALAT | 38 | 37 | 35 | 29 |  |
| HsCRP | 35 | 23 | 27 | 20 |  |
| Body composition |  |  |  |  |  |
| FMI | 33 | - | 28 | - |  |
| VAT | 32 | - | 28 | - |  |
| LMI | 33 | - | 28 | - |  |
| ALMI | 33 | - | 28 | - |  |
| Bone parameters |  |  |  |  |  |
| BMD_LS | 34 | - | 30 | - |  |
| BMD_FN | 34 | - | 30 | - |  |
| BMD_TH | 34 | - | 30 | - |  |
| Low BMD (BMD < -2.0 SD at least at one site) | 14/34 (41) | - | 8/30 (27) | - |  |
| TBS | 34 | - | 28 | - |  |
| P1NP | | 31 | 24 | 22 | 17 |
| β-Crosslaps | | 31 | 23 | 23 | 17 |

Estradiol goal reached and Low BMD are shown as N/Total (percentage). SHBG, Sex hormone-binding globulin. LH, Luteinizing hormone. FSH, Follicle stimulating hormone. BMI, Body mass index. FBG, Fasting blood glucose. HbA1c, Glycated hemoglobin. HOMA-IR, Insulin resistance score. ALAT, Alanine Aminotransferase. HsCRP, High sensitive C-Reactive Protein. FMI, Fat mass index. VAT, Visceral adipose tissue. LMI, Lean mass index. ALMI, Appendicular lean mass index. BMD_LS, Bone mineral density of lumbar spine. BMD_TH, Bone mineral density of total hip. TBS, Trabecular bone score.

**Supplementary Table S2** Effect of Delta E2 (Baseline – 12 months) on metabolic and bone outcomes

|  | **Coefficient** | **Lower** | **Upper** | **SD** | **Z-Value** | **Degree of freedom** | **P-value** |  |
| --- | --- | --- | --- | --- | --- | --- | --- | --- |
| BMI (kg/m^2^) | -0,31 | -0,84 | 0,22 | 0,25 | -1,22 | 20 | 0,237 |  |
| FBG (mmol/L) | -0,02 | -0,10 | 0,07 | 0,04 | -0,45 | 20 | 0,655 |  |
| HbA1c (%) | 0,03 | -0,02 | 0,07 | 0,02 | 1,22 | 21 | 0,236 |  |
| HOMA-IR | -0,11 | -0,40 | 0,18 | 0,14 | -0,81 | 17 | 0,429 |  |
| Triglycerides (mmol/L) | -0,09 | -0,19 | 0,01 | 0,05 | -1,88 | 23 | 0,073 |  |
| HDL-Cholesterol (mmol/L) | 0,00 | -0,03 | 0,04 | 0,02 | 0,26 | 25 | 0,800 |  |
| LDL-Cholesterol (mmol/L) | -0,03 | -0,16 | 0,11 | 0,07 | -0,40 | 26 | 0,694 |  |
| ALAT (UI/L) | -2,30 | -4,97 | 0,36 | 1,30 | -1,78 | 26 | 0,087 |  |
| HsCRP (mg/L) | 0,17 | -0,20 | 0,53 | 0,17 | 0,96 | 18 | 0,349 |  |
| FMI (kg/m^2^) | -0,32 | -0,76 | 0,13 | 0,21 | -1,51 | 17 | 0,149 |  |
| VAT (gr) | -60,97 | -179,55 | 57,61 | 54,77 | -1,11 | 13 | 0,286 |  |
| LMI (kg/m^2^) | 0,02 | -0,20 | 0,23 | 0,10 | 0,16 | 11 | 0,872 |  |
| ALMI (kg/m^2^) | -0,03 | -0,15 | 0,09 | 0,05 | -0,57 | 13 | 0,580 |  |
| BMD_LS (g/cm^2^) | -0,01 | -0,02 | 0,01 | 0,01 | -1,05 | 16 | 0,307 |  |
| BMD_FN (g/cm^2^) | 0,00 | -0,01 | 0,01 | 0,01 | 0,65 | 17 | 0,526 |  |
| BMD_TH (g/cm^2^) | 0,00 | -0,01 | 0,01 | 0,00 | -0,44 | 15 | 0,668 |  |
| TBS | 0,00 | -0,01 | 0,01 | 0,01 | -0,47 | 16 | 0,646 |  |
| P1NP (µg/L) | | 0,38 | -5,30 | 6,06 | 2,73 | 0,14 | 21 | 0,891 |
| β-Crosslaps (ng/L) | | 21,43 | -30,75 | 73,62 | 24,89 | 0,86 | 19 | 0,400 |

Multivariate linear regression adjusted for baseline value, root of administration of GAHT_E2, BMI and age at GAHT initiation. SHBG, Sex hormone-binding globulin. LH, Luteinizing hormone. FSH, Follicle stimulating hormone. BMI, Body mass index. FBG, Fasting blood glucose. HbA1c, Glycated hemoglobin. HOMA-IR, Insulin resistance score. ALAT, Alanine Aminotransferase. HsCRP, High sensitive C-Reactive Protein. FMI, Fat mass index. VAT, Visceral adipose tissue. LMI, Lean mass index. ALMI, Appendicular lean mass index. BMD_LS, Bone mineral density of lumbar spine. BMD_TH, Bone mineral density of total hip. TBS, Trabecular bone score.

**Supplementary Table 3** Effect of reaching E2 goal at 12 months on metabolic and bone outcomes

|  | **Coefficient** | **Lower** | **Upper** | **SD** | **Z-Value** | **Degree of freedom** | **P-value** |  |
| --- | --- | --- | --- | --- | --- | --- | --- | --- |
| BMI (kg/m^2^) | -0,14 | -0,34 | 0,07 | 0,10 | -1,39 | 26 | 0,176 |  |
| FBG (mmol/L) | -0,01 | -0,05 | 0,03 | 0,02 | -0,65 | 18 | 0,525 |  |
| HbA1c (%) | 0,01 | -0,01 | 0,03 | 0,01 | 1,40 | 23 | 0,174 |  |
| HOMA-IR | -0,04 | -0,15 | 0,06 | 0,05 | -0,85 | 21 | 0,404 |  |
| Triglycerides (mmol/L) | -0,03 | -0,07 | 0,01 | 0,02 | -1,48 | 26 | 0,150 |  |
| HDL-Cholesterol (mmol/L) | 0,01 | 0,00 | 0,03 | 0,01 | 1,68 | 28 | 0,105 |  |
| LDL-Cholesterol (mmol/L) | 0,03 | -0,02 | 0,08 | 0,03 | 1,20 | 28 | 0,241 |  |
| ALAT (UI/L) | -0,64 | -1,70 | 0,41 | 0,51 | -1,26 | 28 | 0,220 |  |
| HsCRP (mg/L) | -0,01 | -0,16 | 0,14 | 0,07 | -0,12 | 24 | 0,903 |  |
| FMI (kg/m^2^) | -0,12 | -0,28 | 0,05 | 0,08 | -1,44 | 25 | 0,161 |  |
| VAT (gr) | -11,58 | -47,04 | 23,89 | 17,22 | -0,67 | 25 | 0,508 |  |
| LMI (kg/m^2^) | -0,01 | -0,07 | 0,06 | 0,03 | -0,29 | 22 | 0,778 |  |
| ALMI (kg/m^2^) | -0,02 | -0,06 | 0,02 | 0,02 | -0,86 | 23 | 0,401 |  |
| BMD_LS (g/cm^2^) | 0,00 | 0,00 | 0,00 | 0,00 | -0,18 | 24 | 0,855 |  |
| BMD_FN (g/cm^2^) | 0,00 | -0,01 | 0,00 | 0,00 | -0,65 | 21 | 0,526 |  |
| BMD_TH (g/cm^2^) | 0,00 | 0,00 | 0,00 | 0,00 | -0,49 | 23 | 0,631 |  |
| TBS | 0,00 | 0,00 | 0,00 | 0,00 | -0,65 | 22 | 0,521 |  |
| P1NP (µg/L) | | -0,77 | -3,50 | 1,95 | 1,29 | -0,60 | 17 | 0,557 |
| β-Crosslaps (ng/L) | | 7,25 | -14,61 | 29,10 | 10,46 | 0,69 | 20 | 0,497 |

Multivariate linear regression adjusted for baseline value, root of administration of GAHT_E2, BMI and age at GAHT initiation. SHBG, Sex hormone-binding globulin. LH, Luteinizing hormone. FSH, Follicle stimulating hormone. BMI, Body mass index. FBG, Fasting blood glucose. HbA1c, Glycated hemoglobin. HOMA-IR, Insulin resistance score. ALAT, Alanine Aminotransferase. HsCRP, High sensitive C-Reactive Protein. FMI, Fat mass index. VAT, Visceral adipose tissue. LMI, Lean mass index. ALMI, Appendicular lean mass index. BMD_LS, Bone mineral density of lumbar spine. BMD_TH, Bone mineral density of total hip. TBS, Trabecular bone score.

**Supplementary Figure 1** Multivariate linear regression of change in plasma E2 concentrations and attainment of E2 target with body mass index and plasma triglyceride concentrations


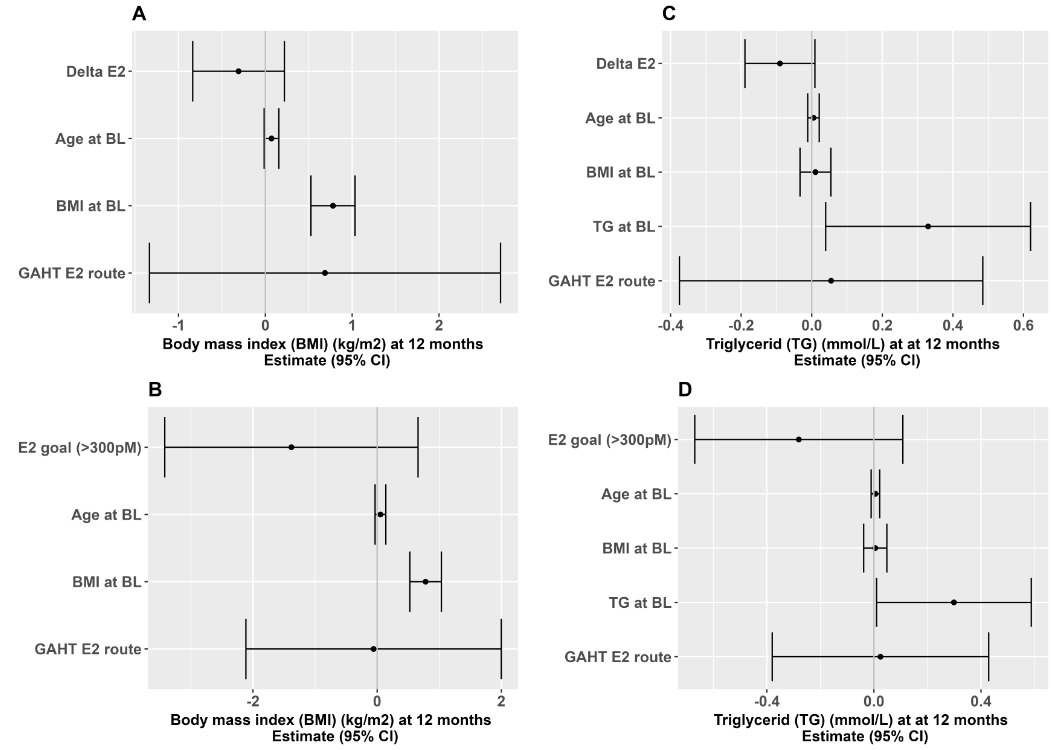


E2, Estradiol. BL, Baseline. BMI, Body mass index. GAHT, Gender affirming hormone therapy. TG, Triglycerides. A) Linear regression of Delta E2 on Body Mass index at 12 months after adjustment for Age at baseline, Body Mass Index at baseline and GAHT E2 route administration. B) Linear regression of Delta E2 on Triglyceride at 12 months after adjustment for Age at baseline, Body Mass Index at baseline, plasma triglyceride concentrations at baseline and GAHT E2 route administration. C) Impact of reaching the E2 goal on Body Mass index at 12 months after adjustment for Age at baseline, Body Mass Index at baseline and GAHT E2 route administration. D) Impact of reaching the E2 goal on Triglyceride at 12 months after adjustment for Age at baseline, Body Mass Index at baseline, Triglyceride at baseline and GAHT E2 route administration. For all panels, brackets show the 95% confidence interval for the association, with the black dote annotating the estimate. The latter corresponds to the absolute expected effect (after adjustment) for each 100 pmol/l increase in E2 levels.
